# Clinical Treatment Guidelines for Tafasitamab plus Lenalidomide in Patients with Relapsed or Refractory Diffuse Large B-Cell Lymphoma

**DOI:** 10.1093/oncolo/oyac256

**Published:** 2023-01-17

**Authors:** Adrienne Nedved, Kami Maddocks, Grzegorz S Nowakowski

**Affiliations:** Department of Pharmacy, Mayo Clinic, Rochester, MN, USA; Arthur G. James Comprehensive Cancer Center, Ohio State University Wexner Medical Center, Columbus, OH, USA; Department of Pharmacy, Mayo Clinic, Rochester, MN, USA

**Keywords:** tafasitamab, lenalidomide, DLBCL, supportive therapy, dose adjustment, routine clinical practice

## Abstract

Diffuse large B-cell lymphoma (DLBCL) accounts for approximately 24% of new cases of B-cell non-Hodgkin lymphoma in the US each year. Up to 50% of patients relapse or are refractory (R/R) to the standard first-line treatment option, R-CHOP. The anti-CD19 monoclonal antibody tafasitamab, in combination with lenalidomide (LEN), is an NCCN preferred regimen for transplant-ineligible patients with R/R DLBCL and received accelerated approval in the US (July 2020) and conditional marketing authorization in Europe (August 2021) and other countries, based on data from the L-MIND study. The recommended dose of tafasitamab is 12 mg/kg by intravenous infusion, administered in combination with LEN 25 mg for 12 cycles, followed by tafasitamab monotherapy until disease progression or unacceptable toxicity. Tafasitamab + LEN is associated with durable responses in patients with R/R DLBCL. The majority of clinically significant treatment-associated adverse events are attributable to LEN and can be managed with dose modification and supportive therapy. We provide guidelines for the management of patients with R/R DLBCL treated with tafasitamab and LEN in routine clinical practice, including elderly patients and those with renal and hepatic impairment, and advice regarding patient education as part of a comprehensive patient engagement plan. Our recommendations include LEN administration at a reduced dose if required in patients unable to tolerate the recommended dose. No dose modification is required for tafasitamab in special patient populations.

## Teaser

It is important for clinicians to understand the optimal use of tafasitamab + lenalidomide and how treatment-emergent toxicities may be managed in the broader population to prevent early treatment discontinuation. This review provides a practical clinical guide for the management of patients with relapsed/refractory diffuse large B-cell lymphoma treated with tafasitamab + lenalidomide in routine clinical practice.

**Implications for Practice**

Tafasitamab + lenalidomide (LEN) is associated with durable responses in patients with relapsed/refractory diffuse large B-cell lymphoma (R/R DLBCL). The majority of clinically significant treatment-related adverse events associated with tafasitamab + LEN are attributable to LEN and can be managed with dose modification and supportive therapy. Here, we provide guidelines for healthcare professionals on the use in routine clinical practice of supportive therapy and dose adjustments during up to 12 cycles of tafasitamab + LEN followed by tafasitamab maintenance monotherapy to facilitate sustained patient benefit in specific populations including the elderly and/or those with renal and hepatic impairment.

## Introduction

Diffuse large B-cell lymphoma (DLBCL) is the most common subtype of non-Hodgkin lymphoma (NHL), accounting for approximately 24% of new cases of B-cell NHL in the US each year, with an age-adjusted incidence rate of 5.6 per 100,000 people per year.^1,2^ Standard first-line treatment option for DLBCL includes 3-6 cycles of R-CHOP (rituximab, cyclophosphamide, doxorubicin, vincristine, and prednisone) chemoimmunotherapy^3^; however, 20-50% of patients relapse or are refractory (R/R) to treatment.^4^

Current treatment options for patients who fail to respond to first-line treatment and are eligible for an aggressive treatment approach include second-line chemotherapy followed by high-dose chemotherapy and autologous stem cell transplant (ASCT) in responding patients.^3^ However, relapse after ASCT eventually occurs in 40-65% of patients.^4^

CD19 is a B-cell-specific transmembrane glycoprotein expressed on the cell surface from the late pro-B to early pre-B cell stages, with increasing expression as B cells mature.^5^ As a central signaling component of cell surface complexes that can include CD21, CD81, and CD225, CD19 links the innate and adaptive arms of the immune system. CD19 is broadly and homogeneously expressed across B-cell malignancies and is able to enhance B-cell antigen receptor signaling, representing an important signaling component for the survival and proliferation of malignant B cells.^5^

Second-line treatments for patients with DLBCL include tafasitamab + lenalidomide (LEN) combination therapy.^3^ Tafasitamab is a humanized, Fc-modified, anti-CD19 monoclonal antibody that enhances antibody-dependent cellular cytotoxicity and phagocytosis with potent in vitro and in vivo activity.^6,7^ LEN, via direct antineoplastic activity, has been shown to enhance natural killer cell-meditated, antibody-dependent cellular cytotoxicity (ADCC) with tafasitamab in vitro.^7,8^ The combination of tafasitamab + LEN for the treatment of R/R DLBCL in transplant-ineligible adults after ≥1 prior therapy was assessed in the L-MIND study^9^ (NCT02399085), receiving accelerated approval in the US (July 2020) and conditional marketing authorization in Europe (August 2021) and other countries.^10-13^

Given that patients treated in clinical practice frequently fall outside the eligibility criteria of a clinical trial, including those at increased risk of hematologic and infectious complications, it is important for clinicians to understand how tafasitamab + LEN may be optimally used and how treatment-emergent toxicities may be managed in this broader population to prevent early treatment discontinuation due to adverse events (AEs). In this review, we discuss a practical clinical guide for the management of patients with R/R DLBCL treated with tafasitamab + LEN in routine clinical practice.

## Methodology

To identify reports of the treatment of patients with R/R DLBCL in clinical trials and routine clinical practice, articles published during 2016–2021 were obtained through MEDLINE and PubMed using the search terms tafasitamab, DLBCL, relapsed, refractory, treatment guideline, and management. Reference lists of selected articles and proceedings of recent hematologic conferences were also reviewed for relevant reports.

## Experience from the L-MIND Study

Response outcomes to tafasitamab + LEN among the broad patient population in our clinical practice have been similar to those reported in the L-MIND study. L-MIND was a multicenter, single arm phase II study assessing tafasitamab + LEN in patients with R/R DLBCL ineligible for high-dose chemotherapy and subsequent ASCT following 1-3 prior systemic treatments.^9^ At a median follow-up of 13.2 months, the objective response rate (ORR) was 60% (48/80 patients, efficacy set), complete response (CR) rate was 43% (34/80 patients), and partial response (PR) rate was 18% (14/80 patients).^9^ Furthermore, 8 patients had DLBCL via transformation of low-grade lymphoma.^14^ Of these 8 patients, 3 had a CR as best response, 4 had a PR, and 1 had stable disease. Although data are limited, patients with transformed lymphoma responded to therapy, indicating efficacy of tafasitamab + LEN in these difficult-to-treat patients.

The most common grade ≥3 treatment emergent adverse events (TEAEs) in L-MIND were neutropenia (48%; 39/81 patients, safety set), thrombocytopenia (17%; 14/81 patients), and febrile neutropenia (12%; 10/81 patients). A total of 37/80 (46%) patients required LEN dose reduction; 62/80 (78%) patients were maintained at a dose of ≥20 mg/day.^9^

Long-term follow-up (≥35 months) of L-MIND reported an ORR of 58%, with a CR rate of 40% and PR rate of 18%.^14^ Median duration of response (DoR) and the exploratory endpoints of median overall survival (OS) and median progression-free survival (PFS) were 43.9 months, 33.5 months, and 11.6 months, respectively. No new or unexpected safety signals were reported during long-term follow-up.^14^ Furthermore, the safety profile of tafasitamab during the extended monotherapy phase was comparable with that of the phase IIa R/R NHL study of tafasitamab monotherapy,^15^ demonstrating limited toxicity for tafasitamab as maintenance therapy.

*Recommendation*: Patients receiving tafasitamab + LEN who are not experiencing symptoms of disease progression should continue combination treatment for a maximum of 12 cycles followed by tafasitamab monotherapy to progression.

## Dosing

The recommended dose of tafasitamab is a 12 mg/kg intravenous infusion,^10,11^ administered in combination with LEN for a maximum of 12 cycles and then as monotherapy until disease progression or unacceptable toxicity (Table 1). Whilst the LEN prescribing information does not specify dosing in combination with tafasitamab for treatment of R/R DLBCL, the tafasitamab prescribing information recommends LEN co-administration at 25 mg/day on days 1-21 for 12 cycles of 28 days each, in line with the LEN indications for multiple myeloma and mantle cell lymphoma.^10,11,16,17^

**Table 1.** Recommended tafasitamab + lenalidomide dosing schedule.

Cycle^a^	Tafasitamab dosing schedule^10,11^	Lenalidomide dosing schedule in L-MIND^9^
Cycle 1	Days 1, 4, 8, 15, and 22	Days 1-21
Cycles 2 and 3	Days 1, 8, 15, and 22	Days 1-21
Cycles 4 to 12	Days 1 and 15	Days 1-21
Cycles 13 to progression	Days 1 and 15	None

^a^Each treatment cycle is 28 days.

In L-MIND, patients with a serum creatinine clearance (CrCL) ≥60 mL/minute received LEN 25 mg on days 1-21 of each 28-day cycle, with stepwise dose reduction permitted in cases of protocol-defined toxicities.^9^ Previous studies of LEN with different dosing regimens in other DLBCL settings have shown varying results with regard to patient outcomes. The Eastern Cooperative Oncology Group (ECOG) ACRIN trial E1412 study indicated a potential clinical benefit of including LEN 25 mg/day on days 1-10 of each R-CHOP cycle, with a 34% reduction in risk of progression or death compared with R-CHOP alone and improved PFS in patients with DLBCL (activated B-cell subtype).^18^ In contrast, the ROBUST study reported no improvement in efficacy when adding LEN 15 mg/day on days 1-14 of each cycle vs R-CHOP alone.^19^

*Recommendation*: A LEN starting dose of 25 mg is recommended in clinical practice (Table 1), except in contraindicated populations for whom dose reduction is recommended, eg, cytopenic patients or those with renal complications (Table 2).

**Table 2.** Recommended dosing adjustments in special patient populations with DLBCL.

	Tafasitamab dose adjustments (dosing schedule shown in Table 1)^10,11^	Lenalidomide dose adjustments (Days 1-21 of each 28-day cycle; authors’ recommendation)
Elderly patients	No dose adjustment required	Stepwise dose reduction (by 5 mg) in patients unable to tolerate a 25 mg dose
Mild hepatic impairment	No dose adjustment required	No dose adjustment required
Mild-to-moderate renal impairment	No dose adjustment required	*Cycles 1-2* CrCl >60 mL/minutes: 25 mg once daily CrCl 30-60 mL/minute: 10 mg once daily
		*Cycles 3-12* CrCl >60 mL/minute: 25 mg once daily CrCl 30-60 mL/minute: 15 mg once daily, if previously tolerated at 10 mg
Severe renal impairment	No data available	*Cycles 1-2* CrCl <30 mL/minute not requiring dialysis: 15 mg every other day CrCl <30 mL/minute requiring dialysis: 5 mg once daily
		*Cycles 3-12* CrCl <30 mL/minute not requiring dialysis: 15 mg every other day CrCl <30 mL/minute requiring dialysis: 5 mg once daily

Abbreviations: CrCL, serum creatinine clearance; DLBCL, diffuse large B-cell lymphoma.

## Tafasitamab + LEN in Special Patient Populations

### Patients with Renal Impairment

Dose reductions for patients with mild-to-moderate renal impairment are not required with tafasitamab.^10,11^ The dose of LEN may need to be reduced in patients with renal impairment (CrCl <60 mL/minute) or after dose interruption for management of increased hepatic transaminases.^16,17^

No data are available regarding use of tafasitamab in patients with severe renal impairment; however, there are no clinically meaningful differences in the pharmacokinetics of tafasitamab in patients with estimated CrCL 30-89 mL/minute. In general, renal elimination of monoclonal antibodies is considered insignificant as their typical molecular weight (150 kDa) is higher than the glomerular filtration threshold (~55 kDa).^20^ The prescribing information for LEN recommends a lower starting dose in patients with renal impairment.^16,17^

*Recommendation*: In patients with renal impairment, LEN dosing adjustments should be made in line with cycle number and CrCl values (Table 2), as previously described in patients with mantle cell lymphoma.^21^

### Elderly Patients

Among 81 patients who received tafasitamab + LEN in L-MIND, 72% were ≥65 years (38% ≥75 years).^10^ Clinical studies were not powered to determine any impact of age on effectiveness. A greater proportion of patients ≥65 years and older (57%) than younger patients (39%) had serious adverse reactions.^10^

Previous investigations of LEN in elderly patients in the first-line setting include a phase I dose escalation trial of short-course LEN in combination with R-CHOP (R2-CHOP) in newly-diagnosed patients with aggressive B-cell NHL (median: 65 years; range: 35-82; 29% ≥75 years).^22^ Here, an escalating dose of 15, 20, and 25 mg/day LEN was administered on days 1-10 only of each 21-day cycle to facilitate hematologic recovery before the next cycle and to allow for synergy with chemotherapy during concomitant therapy.^22^ The results indicated that tolerability was not impacted by age, with no significant differences between patients aged ≥65 years versus <65 years for either hematologic (100 vs 82%, *P* = .2) or non-hematologic toxicities (23 vs 18%, *P* = 1.0).^22^

However, subset analysis of the phase II ECOG ACRIN trial E1412 assessing R2-CHOP with the same LEN dosing schedule (25 mg/day on days 1-10 of each cycle) reported a trend toward more pronounced benefit with the addition of LEN in younger patients (<60 years), alongside a trend toward increased AE burden in older patients (≥60 years).^18^

As a longer treatment course, LEN maintenance monotherapy as 25 mg/day on days 1-21 of a 28-day cycle for 2 years following response to R-CHOP in the phase III REMARC (NCT01122472) study was shown to prolong PFS in patients with DLBCL, with similar outcomes in patients <70 and ≥70 years.^23^

*Recommendation*: Elderly patients should receive a standard dose of tafasitamab + LEN with responses monitored and stepwise dose reduction (5 mg) in patients unable to tolerate 25 mg LEN (Table 2).

### Patients With Mild Hepatic Impairment

Per the prescribing information, no clinically meaningful differences in the pharmacokinetics of tafasitamab have been reported to date in patients with mild hepatic impairment (total bilirubin ≤upper limit of normal [ULN] and aspartate transaminase [AST] >ULN, or total bilirubin 1 to 1.5 times ULN and any AST).^10,11^ The prescribing information for LEN does not advise dose adjustment for patients with mild hepatic impairment; however, a hepatotoxicity warning mandates periodic monitoring with treatment interruption upon elevated liver enzymes and dose reduction following return to baseline values.^16,17^

*Recommendation*: No dose adjustment, per the prescribing information (Table 2).

### Pregnancy

Patients with R/R DLBCL have a median age ≥65 years at diagnosis^2^; however, patients of reproductive age are included within the broader patient profile. Based on its mechanism of action, tafasitamab + LEN may be associated with fetal B-cell depletion, and severe and life-threatening birth defects, including death.^10,11,16,17^ LEN is contraindicated during pregnancy. In alignment with the LEN Risk Evaluation and Mitigation Strategy, females of reproductive potential must avoid pregnancy from at least 4 weeks before to at least 4 weeks after completing LEN therapy by either abstaining from heterosexual sexual intercourse or using 2 methods of reliable birth control.^16^ Two negative pregnancy tests are required prior to initiating therapy and to be repeated during the treatment course. Males must use a condom during sexual contact with females of reproductive potential while taking LEN and for up to 4 weeks after treatment discontinuation. Tafasitamab monotherapy has been shown to be effective in patients with R/R DLBCL, with a favorable safety profile^15^; however, pregnant women were not included in this study and no safety data are currently available on single agent use of tafasitamab during pregnancy.

*Recommendation*: LEN is contraindicated during pregnancy. No safety data are currently available on the use of tafasitamab monotherapy during pregnancy.

## Management of Hematologic Adverse Events

Tafasitamab can result in severe myelosuppression, including neutropenia, thrombocytopenia, and anemia.^10,11^ Dose modifications for tafasitamab and LEN for management of adverse reactions are provided in the tafasitamab prescribing information.^10,11^ Recommendations for management of myelosuppression associated with tafasitamab + LEN include withholding treatment and monitoring complete blood count (CBC) until platelet and neutrophil counts return to ≥50,000/μL and ≥1,000/μL, respectively (Table 3).^10,11,16,17^

**Table 3.** Management of hematologic adverse reactions in patients with DLBCL.

Adverse reaction	Recommended tafasitamab dose modification^10,11^	Recommended lenalidomide dose modification (authors’ recommendation)	Supportive measures (authors’ recommendation)
Neutropenia (ANC ≤1,000/μL for at least 7 days; or ANC ≤1000/μL associated with a body temperature ≥100.4°F [38°C]; or ANC <500/μL)	• Interrupt tafasitamab and monitor CBC weekly until ANC is ≥1000/μL • Resume tafasitamab at the same dose when ANC has returned to an acceptable level	• G-CSF prophylaxis following first occurrence • If neutropenia recurs, interrupt lenalidomide and monitor CBC weekly until ANC is ≥1000/μL • Stepwise 5 mg dose reduction	• G-CSF administration • Pneumocystis pneumonia and antiviral prophylaxis in severely neutropenic patients and those with prior CAR T-cell therapy • Consider antibacterial, antiviral, and antifungal prophylaxis in patients with prolonged neutropenia (ANC ≤500/μL for at least 7 days) or those at increased risk due to previous neutropenic complication or recurring episodes of neutropenic fever
Thrombocytopenia (platelet count ≤50 000/μL)	• Interrupt tafasitamab and monitor CBC weekly until platelet count is ≥50 000/μL • Resume tafasitamab at the same dose when platelet count has returned to an acceptable level	• Interrupt lenalidomide and monitor CBC weekly until platelet count is ≥50 000/μL • Stepwise 5 mg dose reduction	• Monitor for signs and symptoms of active bleeding while on venous thromboembolism prophylaxis for lenalidomide • Transfuse platelets, if necessary, per standard institutional practice

Abbreviations: ANC, absolute neutrophil count; CAR, chimeric antigen receptor; CBC, complete blood count; DLBCL, diffuse large B-cell lymphoma; G-CSF, granulocyte colony stimulating factor.

In L-MIND, neutropenia was the most common all-grade AE at 1-year follow-up (median follow-up: 13.2 months), occurring in 49% of patients, and the most common grade ≥3 AE, occurring in 48% of patients.^9^ Neutropenia was managed with granulocyte colony stimulating factor (G-CSF) in 44% of patients, the majority of whom recovered to baseline neutrophil counts within 1 week.^9^

G-CSF prophylaxis is widely used to reduce the incidence and severity of chemotherapy-induced neutropenia by promoting the production of granulocytes or antigen presenting cells.^24^ Whilst G-CSF is generally not recommended within 24 hours of conventional chemotherapy, to allow metabolism and excretion time for most cytotoxic drugs, growth factors could be prescribed in L-MIND during the treatment and follow-up periods at the investigator’s discretion. As a targeted immunotherapy, tafasitamab mediates ADCC and antibody-dependent cellular phagocytosis, and exerts direct cytotoxicity on CD19-expressing B-cells^10^; hence G-CSF prophylaxis can be given in the same visit as tafasitamab dosing. Similarly, G-CSF administration as secondary prophylaxis can overlap with LEN administration.

Pneumocystis pneumonia prophylaxis is based on previous therapy. For example, post chimeric antigen receptor (CAR) T-cell therapy, patients are at risk of infection during the post-transplant period as a consequence of prolonged cytopenia following depletion of normal CD19-expressing B-cells.^25^ CAR T-cell therapy may result in profound immune deficits that may persist for several years, warranting antiviral (acyclovir or valacyclovir) prophylaxis concomitant with subsequent lines of therapy. Other infectious complications are not typically monitored in routine practice unless there is a previous history of infection or evidence of neutropenia.

CBC should be monitored prior to and throughout each treatment cycle.^10,11,16,17^ Patients with neutropenia should be monitored for signs of infection and advised to report signs or symptoms of bleeding or bruising immediately, especially when using concomitant medication that may increase bleeding risk.

*Recommendation 1*: In patients with myelosuppression (neutropenia [absolute neutrophil count ≤1,000/μL for at least 7 days; absolute neutrophil count ≤1,000/μL associated with a body temperature ≥100.4°F (38 °C); or absolute neutrophil count <500/ μL], or thrombocytopenia [platelet count ≤50,000/μL]), tafasitamab should be interrupted; dosing may restart at the same dose when neutrophil/platelet count has returned to an acceptable level (Table 3). If neutropenia recurs despite G-CSF prophylaxis or in case of thrombocytopenia, LEN should be interrupted and resumed with a stepwise 5 mg dose reduction.

*Recommendation 2*: Patients with prolonged neutropenia (absolute neutrophil count ≤500/μL for at least 7 days) or those at increased risk due to previous neutropenic complication or recurring episodes of neutropenic fever may receive antibacterial, antiviral, and antifungal prophylaxis (Table 3).

## Management of Non-hematologic Adverse Events

In our experience, infusion-related reactions (IRRs) with tafasitamab + LEN are uncommon in clinical practice. In L-MIND, IRRs (all grade 1) occurred in 6% of patients, 80% of which occurred during cycle 1 and 2, with reported symptoms of fever, chills, rash, flushing, dyspnea, and hypertension.^9-11^ Discontinuation of tafasitamab was not required. Hypersensitivity reactions such as angioedema, anaphylaxis, and anaphylactic reactions have been reported with LEN.^16,17^

Pre-treatment with medications such as acetaminophen, histamine H1 or H2 receptor antagonists, and/or glucocorticosteroids is recommended 30-120 minutes prior to tafasitamab infusion, or following standard institutional guidelines for monoclonal antibodies to minimize IRRs.^9^ Pre-treatment is optional for patients not experiencing an IRR during the first 3 infusions.^10^

*Recommendation*: Recommendations for management of IRRs associated with tafasitamab include temporary interruption of tafasitamab infusion to manage signs and symptoms (grades 2-3), and, in the case of severe IRR (grade 4), discontinuation of tafasitamab (Table 4). In patients who have experienced an IRR during a prior treatment cycle, pre-medication is recommended before each subsequent infusion.^10^

**Table 4.** Management of IRRs adverse reactions in patients with DLBCL.

Adverse reaction	Recommended tafasitamab dose modification^10,11^	Supportive measures^10,11^
Grade 2 IRR	• Interrupt tafasitamab infusion immediately and manage signs and symptoms • Once symptoms resolve or reduce to grade 1, resume infusion at ≤50% of the rate at which the reaction occurred • If no further reaction within 1 hour and vital signs are stable, the infusion rate may be increased every 30 minutes as tolerated, to the rate at which the reaction occurred	• Pre-treatment with acetaminophen, histamine H1 receptor antagonists, histamine H2 receptor antagonists, and/or glucocorticosteroids is recommended 30-120 minutes prior to tafasitamab infusion, or following standard institutional guidelines for monoclonal antibodies - Pre-medications can be adjusted for infusion reaction management: acetaminophen (1000 mg or equivalent), diphenhydramine (25-50 mg IV or equivalent), famotidine (20 mg or equivalent), methylprednisolone (80-120 mg IV or equivalent) - If a medication is not used from this list empirically, and a reaction occurs, that medication can be added in for secondary prophylaxis on subsequent infusions - Any of the pre-medications may be repeated per institution discretion as required for infusion reaction management • For patients who experienced an IRR during a prior treatment cycle, pre-treatment is recommended before each subsequent infusion - Pre-treatment is optional for subsequent infusions in patients not experiencing an IRR during the first 3 infusions • Additional supportive care including but not limited to, epinephrine, IV fluids, vasopressors, oxygen, bronchodilators, may be used to manage infusion reactions • Meperidine 25 mg may also be included as an option for rigors or chills • Montelukast has been studied as a premedication with other monoclonal antibodies and may be considered for prevention of subsequent tafasitamab infusion reaction
Grade 3 IRR	• Interrupt tafasitamab infusion immediately, and manage signs and symptoms • Once symptoms resolve or reduce to grade 1, resume infusion at ≤25% of the rate at which the reaction occurred • If the patient does not experience further reaction within 1 hour and vital signs are stable, the infusion rate may be increased every 30 minutes as tolerated to a maximum of 50% of the rate at which the reaction occurred • If the reaction returns after re-challenge, stop the infusion immediately	
Grade 4 (life-threatening)	• Stop the infusion immediately and permanently discontinue tafasitamab	

Abbreviations: DLBCL, diffuse large B-cell lymphoma; IRR, infusion-related reaction; IV, intravenous.

## Management of Other AEs Associated with LEN

Severe cutaneous reactions, including Stevens-Johnson syndrome, toxic epidermal necrolysis, and drug reaction with eosinophilia and systemic symptoms have been reported with LEN.^16,17^ In patients with follicular lymphoma or marginal zone lymphoma, LEN in combination with rituximab resulted in tumor lysis syndrome and tumor flare reactions (TFR) in 1.1% and 10.8% patients, respectively.

Other AEs related to LEN include thromboembolic events, allergic reactions/hypersensitivity, and rash.^9^ Deep vein thrombosis (DVT) (7.4%), pulmonary embolism (PE) (3.7%), and stroke (2.3%) have been reported in patients with multiple myeloma receiving LEN and dexamethasone^16,17^; similarly, in L-MIND, grade ≥3 DVT (1%) and PE (4%) were observed in patients with R/R DLBCL.^9^

*Recommendation 1*: In routine clinical practice, thromboprophylaxis is recommended^16,17^; options include aspirin, low molecular weight heparin, or new oral anticoagulants such as apixaban and rivaroxaban (Table 5).

**Table 5.** Dose modification during other adverse reactions.

Adverse reaction	Severity	Recommended lenalidomide dose modification^16,17^
Thromboembolic events	Any	• Interrupt dosing • Start therapeutic anticoagulation per local guidelines
	Following stabilization	• Restart at original dose at investigator’s discretion • Continue anticoagulation throughout the course of lenalidomide treatment
Severe cutaneous reaction	Grades 2-3	• Consider interruption or discontinuation
	Grade 4	• Permanently discontinue treatment
Allergic reaction or hypersensitivity	Any angioedema, anaphylactic reaction, or exfoliative or bullous rash, or if SJS, TEN, or DRESS is suspected	• Permanently discontinue treatment
	Patients with a history of severe rash associated with thalidomide	• Contraindicated
	Other forms of skin reaction	• Consider interruption or discontinuation, depending on severity

Abbreviations: DRESS, drug reaction with eosinophilia and systemic symptoms; SJS, Stevens-Johnson syndrome; TEN, toxic epidermal necrolysis.

*Recommendation 2*: LEN dose reduction due to AEs should follow a stepwise pattern; where a 5 mg dose is not tolerated, we recommend discontinuation of LEN and continuation with tafasitamab monotherapy.

*Recommendation 3*: LEN should be temporarily interrupted or discontinued for grades 2-3 skin rash and permanently discontinued for grade 4 rash and severe cutaneous reactions (Table 5). For TFR, LEN treatment may be continued with grade ≤2 TFR at the physician’s discretion; however, for grade ≥3 AEs it is recommended to withhold LEN treatment until TFR resolves to grade ≤1.

## Tafasitamab + LEN and Other Approved Therapies in R/R DLBCL

Both tafasitamab + LEN and the anti-CD19 CAR T-cell therapy axicabtagene ciloleucel (axi-cel) are licensed for the treatment of patients with DLBCL after one prior line of therapy (Table 6): tafasitamab + LEN is indicated for the second-line treatment of patients who are ineligible for ASCT^10^ and axi-cel received a revised indication in April 2022 for second-line use in adult patients with large B-cell lymphoma with primary refractory disease or who relapse within 12 months of first-line chemoimmunotherapy.^26^ Axi-cel and the CAR T-cell therapies tisagenlecleucel and lisocabtagene maraleucel,^26,27,28^ the oral nuclear transport (XPO1) inhibitor selinexor,^29^ the antibody-drug conjugates polatuzumab vedotin and loncastuximab tesirine^30,31^ are approved as third-line treatments. CAR T-cell therapy is an effective treatment option for some patients with NHL, particularly those with R/R DLBCL^32^; however, this therapy must be administered at a specialized treatment center and is associated with a high toxicity burden, as well as the time and costs associated with the manufacture and delivery of CAR T cells.^33-35^

**Table 6.** Treatment options for patients with DLBCL after one or more prior lines of therapy.

Drug	Indication	Line of therapy
Tafasitamab + lenalidomide^11^	Adult patients with R/R DLBCL not otherwise specified, including DLBCL arising from low grade lymphoma, and who are not eligible for ASCT	≥2nd line
Axicabtagene ciloleucel^26^	Adult patients with large B-cell lymphoma that is refractory to first-line chemoimmunotherapy or that relapses within 12 months of first-line chemoimmunotherapy	≥2nd line
	Adult patients with R/R DLBCL after 2 or more lines of systemic therapy, including DLBCL not otherwise specified, primary mediastinal large B-cell lymphoma, high grade B-cell lymphoma, DLBCL arising from follicular lymphoma, and adult patients with R/R follicular lymphoma	≥3rd line
Polatuzumab vedotin + rituximab + bendamustine (pola-BR)^30^	Adult patients with R/R DLBCL, not otherwise specified, after at least 2 prior therapies	≥3rd line
Tisagenlecleucel^27^	Adult patients with R/R DLBCL after 2 or more lines of systemic therapy including DLBCL not otherwise specified, high grade B-cell lymphoma and DLBCL arising from follicular lymphoma	≥3rd line
Lisocabtagene maraleucel^28^	Adult patients with R/R DLBCL after 2 or more lines of systemic therapy, including DLBCL not otherwise specified (including DLBCL arising from indolent lymphoma), high-grade B-cell lymphoma, primary mediastinal large B-cell lymphoma, and follicular lymphoma grade 3B	≥3rd line
Selinexor^29^	Adult patients with R/R DLBCL not otherwise specified, including DLBCL arising from follicular lymphoma	
Loncastuximab tesirine^31^	Adult patients with R/R DLBCL after 2 or more lines of systemic therapy, including DLBCL not otherwise specified, DLBCL arising from low grade lymphoma, and high-grade B-cell lymphoma	

Abbreviation: ASCT, autologous stem cell transplant; R/R DLBCL, relapsed/refractory diffuse large B-cell lymphoma.

DoR, PFS, and OS appear to be improved in patients who receive tafasitamab + LEN as second-line therapy for R/R DLBCL, compared with more heavily pre-treated patients,^14^ however there are as yet no studies to assess relative effectiveness of different anti-CD19 treatment sequences. Changes in CD19 expression after tafasitamab treatment may impact subsequent CD19-targeted approaches; understanding expression changes could inform optimal sequencing of treatment options.

The potential for functional interference between tafasitamab and CD19-directed CAR T cells has been discussed in the literature. In clinical practice, case studies for 2 patients refractory to tafasitamab + LEN reported tumor biopsies with no detectable CD19 26 and 7 days after last tafasitamab dose.^36^ Subsequent biopsies at 32 and 25 days post-tafasitamab, respectively, showed re-emergence of CD19 expression for these patients, leading the authors to suggest that sustained antigen blockade from a prior anti-CD19 treatment may delay subsequent CD19-targeted therapy.^36^

CD19 expression was also assessed in tumor biopsies from a subset of patients with R/R DLBCL before and after tafasitamab treatment in the L-MIND study.^37^ Of 8 lymph node biopsies taken after tafasitamab treatment, 3 were taken within 85 days (or 5 tafasitamab half-lives) of last treatment with tafasitamab, and 5 were taken at >85 days. Immunohistochemical analysis showed comparable CD19 expression before and after tafasitamab therapy. DNA and RNA analyses did not find evidence for CD19 mutations, dominant exon skipping, or loss of CD19 mRNA expression indicative of resistance to further CD19-targeted therapy, indicating maintenance of CD19 expression after tafasitamab therapy.

Long-term follow-up of the L-MIND study revealed outcomes in 2 patients who received CAR T-cell therapy after tafasitamab + LEN and tafasitamab monotherapy to disease progression.^14^ One patient received 8 cycles of tafasitamab + LEN, achieving a PR before experiencing disease progression. They did not respond to further chemotherapy or CAR T-cell therapy and died 4 months after CAR T-cell treatment.^14^ The second patient had stable disease to 6 cycles of tafasitamab + LEN as third-line treatment followed by eventual progression.^14,38^ They subsequently received 4 cycles of R-GemOx chemotherapy followed by CAR T-cell therapy, with CR achieved 1 month after CAR T-cell therapy.^38^ This patient maintained a CR for 1 year and then died due to acute myeloid leukemia 2 years post-CAR T-cell therapy. Given the ~16 day half-life of tafasitamab, the authors of this case study suggest it was likely eliminated prior to subsequent anti-CD19 therapy.^38^ The authors posited that CD19 antigen escape did not account for relapse in this patient, since they achieved sustained remission for nearly 1 year with anti-CD19 CAR T-cell therapy. This suggests that efficacy of CAR T-cell therapy may potentially be sustained in the clinical setting when used after tafasitamab + LEN treatment.

These results are consistent with previous analysis of CD19 expression in patients with R/R DLBCL after treatment with loncastuximab tesirine and subsequent CD19-directed CAR T-cell therapy.^39^ Fourteen patients received a median of 2 cycles (range, 1-7) of loncastuximab tesirine: 8 patients had refractory disease, 5 patients had a PR, and 1 patient had a CR. All responding patients had disease progression before proceeding with CAR T-cell therapy, with a median interval of 120 days (range, 22 to 600) between loncastuximab tesirine and CAR T-cell therapy. Six patients received additional lines of therapy between loncastuximab tesirine and CAR T-cell treatment (median 1; range, 1-3). Immunohistochemical analysis of repeat biopsies from 10 patients after loncastuximab tesirine and before CAR T-cell administration showed CD19 expression in all 10 patients. The 4 patients with unknown CD19 expression status after loncastuximab tesirine achieved CR with anti-CD19 CAR T-cell therapy, thus it was considered unlikely that these patients had relapsed with CD19-negative disease.

*Recommendation*: Whilst further studies are required to establish the persistence of CD19 expression across anti-CD19 modalities to guide sequencing of therapies following initial anti-CD19 therapy, these reports suggest that prior anti-CD19 treatment does not preclude a subsequent response to anti-CD19 CAR T-cell therapy.

## Supporting Patients on Long-Term Treatment

Appropriate use of supportive therapy and dose adjustments to facilitate administration of up to 12 cycles of tafasitamab + LEN, followed by tafasitamab monotherapy as maintenance treatment, may support patient adherence to treatment and thus facilitate a favorable long-term safety and efficacy profile with this treatment combination.^9,14^

Patient education is an effective tool to improve medication adherence (Table 7), as is a pragmatic approach to treatment scheduling. Experience shows us that a patient who feels that special events in daily living can be accommodated by an understanding healthcare team is more likely to adhere to treatment in the long term. To optimize patient education, it is important to explore the patient perspective: this is associated with greater patient satisfaction, which has been shown in various oncology settings to be associated with survival outcomes.^40^

**Table 7.** Patient education.

Patients should be made aware at diagnosis of DLBCL of the possibility for successive lines of therapy.
Patients should be informed that tafasitamab + lenalidomide may be associated with durable responses in patients with R/R DLBCL.
Patients should be informed at the start of treatment that the dosing schedule for tafasitamab + lenalidomide requires weekly clinic visits during the first 3 treatment cycles, plus an additional visit on day 4 of cycle 1, and that this reduces to fortnightly visits thereafter until disease progression.
Patients should be advised that tafasitamab + lenalidomide is a chemotherapy-free treatment option that works by binding to the CD19 receptor on the surface of malignant (abnormal) B cells to help the body to slow or stop their growth.
Patients should be informed that the majority of clinically significant treatment-associated adverse events are attributable to lenalidomide and can be managed with dose modification and supportive therapy.
Patients of reproductive potential must be advised to use contraception during treatment with tafasitamab + lenalidomide and for up to 3 months after the last dose.

Abbreviation: R/R DLBCL, relapsed/refractory diffuse large B-cell lymphoma.

## Summary

The combination of tafasitamab + LEN has a well-defined safety profile and is an NCCN preferred regimen for transplant-ineligible patients with R/R DLBCL who relapse >12 months after first-line therapy. LEN-induced toxicities can be managed in most patients with dose modification and supportive therapy, including growth factor support. Per the prescribing information, no clinically meaningful differences in the pharmacokinetics of tafasitamab have been observed to date based on age (16-90 years), mild-to-moderate renal impairment, or mild hepatic impairment^10,11^; no dose adjustment for tafasitamab is required in these populations (Table 2).

Currently, most therapies for R/R DLBCL have a fixed therapy duration. Tafasitamab + LEN is novel in that tafasitamab monotherapy is given until progression. Whether a fixed duration of tafasitamab monotherapy following combination treatment would offer the same benefits as treatment until progression is an area for future research. Furthermore, reduced frequency of tafasitamab infusions from bi-weekly to monthly is being investigated in the ongoing MINDway study (NCT05222555). If the clinical benefit observed in L-MIND can be maintained and optimized by reducing treatment frequency and burden, additional improvements in patient quality of life and reduced healthcare utilization could be realized.

## Data Availability

No new data were generated or analyzed in support of this research.
